# The effect of Er:YAG and Nd:YAG lasers and total-etch and universal adhesives on composite resin restoration microleakage

**DOI:** 10.4317/jced.62641

**Published:** 2025-05-01

**Authors:** Farahnaz Sharafeddin, Arefeh Torabi Parizi, Maryam Jamshidi

**Affiliations:** 1 Department of Operative Dentistry, Biomaterials Research Center, School of Dentistry, Shiraz University of Medical Sciences, Shiraz, Iran

## Abstract

**Background:**

The present investigation examined Er:YAG and Nd:YAG lasers efficiency in reducing microleakage when paired with two adhesive systems.

**Material and Methods:**

Class V cavities were created on the buccal and lingual sides of 30 extracted premolars. The samples were distributed into the following groups: 1) 37% phosphoric acid + Adper Single Bond 2; 2) Nd:YAG laser + Adper Single Bond 2; 3) Er:YAG laser + Adper Single Bond 2; 4) 37% phosphoric acid + Gluma Universal Bonding; 5) Nd:YAG laser + Gluma Universal Bonding; 6) Er:YAG laser + Gluma Universal Bonding. Each specimen was cured and restored with nanohybrid composite Z350. Dye penetration was used to assess microleakage and examined under a stereomicroscope. The findings were compared using Mann-Whitney, Wilcoxon Signed Ranks, and Kruskal-Wallis tests (α = 0.05).

**Results:**

The microleakage score in the occlusal margin was comparable between the groups. On the other hand, the microleakage in the gingival margin was significantly different between the groups (*P*=0.004). The post-hoc comparison showed that the microleakage score for samples treated with Er: YAG was significantly lower than those treated with Nd: YAG (*P*<0.05). The microleakage score in the gingival margin was significantly higher than that of the occlusal margin in all groups (*P*<0.05), except for the Er: YAG + Single Bond 2 group (*P*=0.15).

**Conclusions:**

Except for Er:YAG + Single Bond 2, all groups showed more microleakage at the gingival margin compared to the occlusal margin. The Er:YAG laser outperformed the Nd:YAG laser at the gingival margin.

** Key words:**Dental Adhesives, Dental Leakage, Er-YAG Laser, Nd-YAG Laser.

## Introduction

Significant progress has been made in the introduction of lasers, and their potential uses have greatly expanded in dentistry (1). Lasers can cause surface changes that may impact the microleakage of adhesive restorations. Some research suggests that laser treatment of the dentin can improve the effectiveness of adhesive restorations (2,3). Laser irradiation alters dental hard tissue’s physical and chemical characteristics (4). It affects the microstructure of the dentin by making a rough surface with open tubules and causing mild demineralization, which improves composite adhesion (5). Additionally, several authors have proposed that pretreating the enamel and dentin with lasers may improve the bonding of resin composite restorations and reduce microleakage (6,7).

Erbium lasers are often employed for dental treatments that involve hard tissue since their wavelengths are absorbable by the enamel and dentin’s hydroxyapatite and water (8,9). Er:Yag laser treatment causes dentin surfaces to become irregular, and dentinal tubules become exposed, enhancing the bond between the dentin and adhesive (10). Lasers are valuable for the enamel and dentin conditioning before adhesive procedures (11). The Nd:YAG laser is more commonly employed for treatments involving soft tissue. Nd:YAG laser manufacturers claim that their products can prepare enamel and dentin surfaces for bonding. Prior morphological investigations have demonstrated that the Nd:YAG laser may change dental hard tissues by melting and recrystallizing their surfaces. These changes result in a molten surface with honeycomb-like irregularities or scattered craters, providing an ideal surface for the mechanical bonding of the resin (12,13).

The adhesion between the substrate and the restoration is essential in influencing the restorations’ lifespan. Dentin adhesives are vital in achieving optimal marginal sealing for resin composites. Poor adaptation can result in microleakage, which leads to sensitivity, recurrent caries, pulp irritation, and, eventually, treatment failure (14,15). The universal adhesives has enabled dentists to utilize a single adhesive with a variety of bonding procedures, including “self-etch,” “etch-and-rinse,” and “selective etch.” They adhere to different substrates, both direct and indirect restorations (16).

Few studies explored Er:YAG and Nd:YAG lasers effect on composite resin restorations’ microleakage. A study found that the Er:YAG laser did not influence the microleakage. However, the CO2 laser increased the microleakage (6). However, they only investigated a total-etch adhesive. There is a limited number of research on how lasers interact with universal adhesives. As a result, this study investigates the influence of Er:YAG and Nd:YAG lasers, as well as total-etch and universal adhesives, on composite resin restorations’ microleakage. The first null hypothesis asserts that laser preparation of cavity surfaces following acid etching has no substantial effect on microleakage in composite restorations. The second null hypothesis questions whether universal adhesives perform differently from total-etch adhesives.

## Material and Methods

This study was performed under the ethics committee approval number IR.SUMS.DENTAL.REC.1400.102.

-Sample preparation

Thirty intact human premolars removed for orthodontic treatment were gathered. The samples were kept at 4°C in a 0.1% thymol suspension with a pH of 7 for 30 days. The samples were then rinsed for 60 s before air-drying. Each tooth was mounted in acrylic cubes that were 2 cm long, 1.5 cm high, and 2 cm wide, with the mount 3 mm beneath the cemento-enamel junction. Standard Class V cavities (5 mm length, 3 mm width, and 2 mm depth) were created on both the lingual and buccal sides using a high-speed bur and air spray. The cavity’s occlusal and gingival margins were placed in the enamel and 1 mm beneath the CEJ, respectively. A new bur for preparing each five cavities.

-Sample allocation

**Figure 1:**
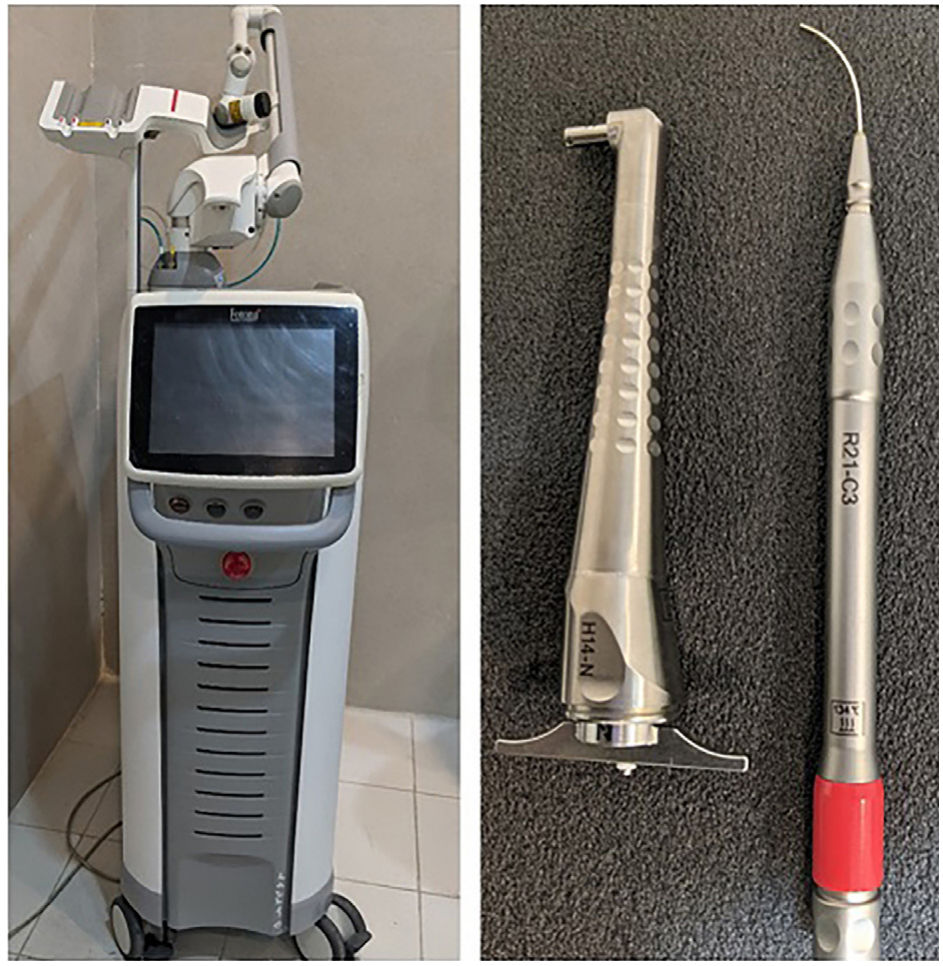
Fotona laser with Er:YAG and Nd:YAG lasers tip.

Sample allocationThe samples were randomly distributed into 6 groups (n=5):

• Group 1: Dentin was etched for 15 s with 37% phosphoric acid (Etch One, Nik Darman, Iran) and rinsed for 20 s. Excess moisture was removed by cotton pellets. The bonding system, Adper Single Bond 2 (3M ESPE, USA), was applied to the surface in two layers for 15 s and the solvent was evaporated.

• Group 2: Nd:YAG laser (LightWalker AT, Fotona, Ljubljana, Slovenia) equipped with fiber optic handpiece model R21-C2 was used in a freehand, non-contact mode for 60 seconds on the tooth surface (17). The laser irradiated perpendicularly at the sample at a distance of 1 mm at a rate of 1 mm s, with water rinsing at four ml/s. The Adper Single Bond 2 was then applied as described in Group 1.  Table 1 shows details on the study materials and laser settings.

• Group 3: The Er:YAG laser (LightWalker AT, Fotona, Ljubljana, Slovenia) with fiber optic type H14-N was used at a speed of 1 mm/s and water rinsing at 4 ml/s for 60 seconds within the cavity (17). The laser beam was positioned perpendicular to the sample at a distance of 1 mm. The bonding system, Adper Single Bond 2, was used in the same manner as in Group 1.

• Group 4: Dentin was etched for 15 s, then rinsed from 1 cm away for 20 s. Cotton pellets were employed to remove extra water. The universal bonding system (Kulzer-Gluma, Germany) was then applied in two layers for 20 s, followed by 5 s of moderate air blast to evaporate the solvent.

• Group 5: The Nd:YAG laser (LightWalker AT, Fotona, Ljubljana, Slovenia) was used in a freehand, non-contact mode for 60 s at a speed of 1 mm/s and water rinsing at 4 ml/s, similar to Group 2. The laser beam was aimed perpendicularly at a distance of 1 mm. Gluma universal bonding was then applied, as detailed in Group 4.

• Group 6: Er:YAG laser (LightWalker AT, Fotona, Ljubljana, Slovenia) was used at 1 mm/s and water rinsing at 4 ml/s for 60 s in a cavity. The laser beam was positioned perpendicularly at a distance of 1 mm. Then, Gluma universal adhesive was used, as stated in Group 4. Figure 1 illustrates both theEr:YAG and Nd:YAG lasers

The specimens were cured for 10 s using a 1200 mW/cm² LED curing lamp (Demi Plus, Kerr, Switzerland) at a wavelength of 470 nm. A nano-hybrid composite (Z350, 3M ESPE, USA, Dentin A2) was used to restore the cavities. After incubation in distilled water at 37°C for 24 h, the restorations were polished using OptiDisc (Kerr, USA). The samples underwent 1000 thermocycling cycles (PC300; Vafaei, Iran) with 30 s dwell periods in water baths set at 5°C and 55°C.

-Evaluating margin microleakage

**Figure 2:**
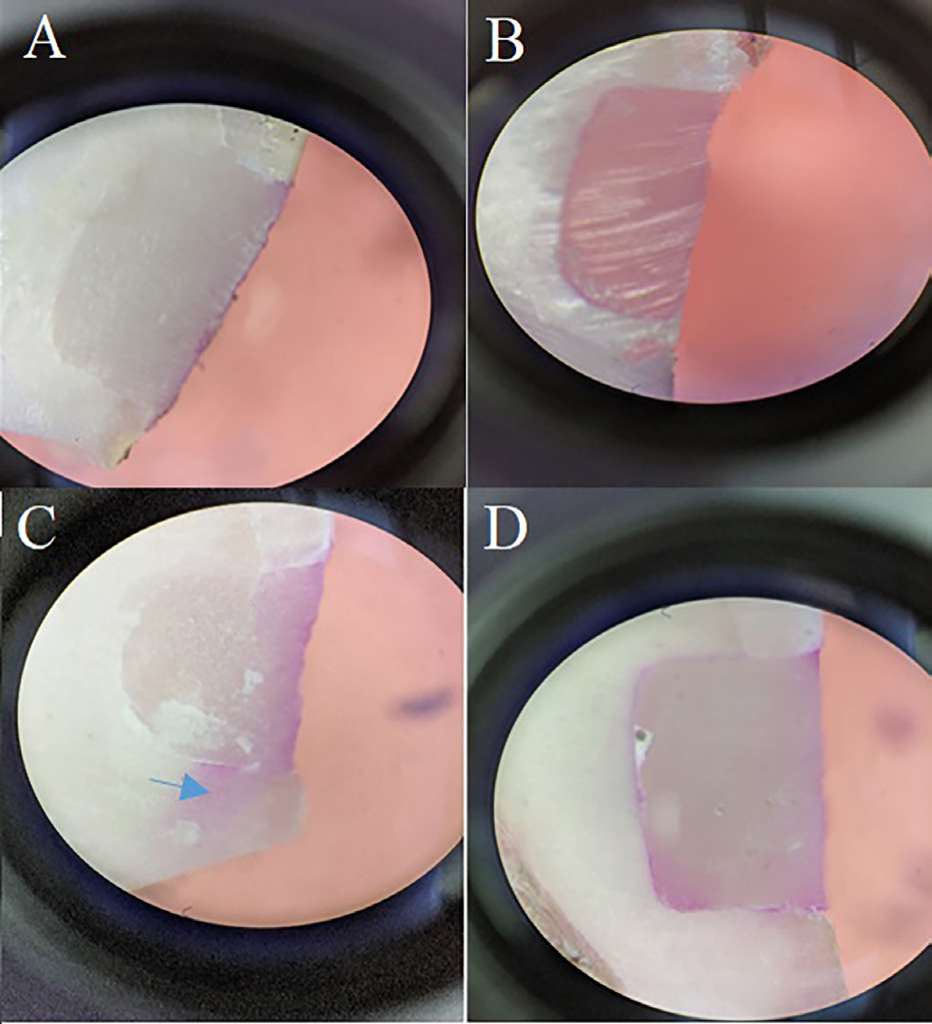
Stereomicroscope Image (×40) of Dye Penetration scores; the arrow shows dye penetration. The occlusal margin is at the top and the gingival margin is at the bottom of each image (A) 0 in occlusal margin and 0 in gingival margin (Single Bond 2) (B) 0 in occlusal margin and 0 in gingival margin (Er:YAG+Single bond 2) (C) 0 in occlusal margin and 3 in gingival margin (Er:YAG+Gluma) (D) 1 in occlusal margin and 3 in gingival margin (Gluma).

The dental surfaces were painted with green nail polish up to 1 mm from the margin. The samples were incubated in a 2% fuchsin solution (Merck, Germany) at 37°C for 24 h. Then, they were rinsed thoroughly. The samples were sectioned horizontally and vertically under water coolant with a cutting machine (Demco E96, CMP Industries, NY, USA) and a diamond disk (D & Z, Germany). A stereomicroscope (BestScope BS-3060C, China) was used at 40x magnification to rate dye penetration. A blinded operator evaluated the microleakage according to the following criteria according to dye penetration extend (18): Score 0 (no penetration), Score 1 (penetration < halfway up the axial wall), Score 2 (penetration > halfway up the axial wall), and Score 3 (penetration along occlusal and axial walls), (Fig. 2).

-Statistical analysis

The findings were compared using Kruskal-Wallis and Mann-Whitney, and Wilcoxon Signed Ranks tests (α=0.05).

## Results

According to  Table 2, the microleakage score in the occlusal margin was comparable between the groups. On the other hand, the microleakage in the gingival margin was significantly different between the groups (*P*=0.004). The post-hoc comparison showed that the microleakage score for samples treated with Er: YAG was significantly lower than those treated with Nd: YAG (*P*<0.05). The microleakage score in the gingival margin was significantly higher than that of the occlusal margin in all groups (*P*<0.05), except for the Er: YAG + Single Bond 2 group (*P*=0.15).

## Discussion

Researchers have identified the Er:YAG laser potential in ablating dental structures, making it a valuable tool for removing caries, preparing cavities, and altering dentin and enamel surfaces before restoring them with adhesive materials (19-21). A comparison between cavities prepared using a bur or the Er:YAG laser found that cavities treated with acid etching before bonding exhibited the least microleakage at the occlusal margin (19). In a study where dentin was acid-etched, composite sealing proved to be more effective than Er:YAG laser treatment. In their research, the dentin was irradiated for 20 seconds at 140 mJ and 4 Hz with the Er:YAG laser, and the ScotchBond Multipurpose adhesive system was used (20). In our study, however, the Er:YAG laser parameters were set at 200 mJ and 20 Hz for 60 seconds, which could explain the more favorable results observed. Additionally, the adhesive used in the previous study was a fourth-generation system, which generally shows better outcomes than the fifth-generation simplified and universal adhesive used in our study.

Another study showed that coronal and root dentin cavity preparations using the Er:YAG laser resulted in less microleakage. Scanning electron microscope (SEM) observations in their study revealed that laser-treated cavities were irregular and free from the smear layer. This allowed for better primer and adhesive infiltration, reducing microleakage (21). The current study showed no difference between the Er:YAG laser groups, whether using a total-etch or universal adhesive, at the occlusal margin. This suggests that Er:YAG laser pretreatment may not impair marginal sealing and could offer results comparable to acid etching. At the gingival margin, while no differences were found between the Er:YAG groups and those using Single Bond or Gluma, the overall results indicated reduced microleakage compared to other groups. These findings suggest that the Er:YAG laser can effectively prepare the dentin for both adhesive systems.

Bonding agents were developed to modify or eliminate the smear layer created by rotary equipment. Because laser pretreatment of dentin leaves no smear layer, bonding chemicals may have a lower effect on laser-treated dentin, emphasizing the necessity for bonding agents formulated particularly for laser-ablated surfaces. Nonetheless, our findings showed that bonding agents may still operate well following laser pretreatment.

In one study, it was reported that the Nd:YAG laser energy and frequency influenced its interaction with dentin surfaces. The best results were observed with 1 W power and 15 Hz frequency, showing reduced microleakage compared to the group etched with 35% phosphoric acid. In that study, the adhesive system was Single Bond 2 (22). The 1 W and 10 Hz parameters used in our study, which were similar to theirs, showed inferior results to 1 W and 15 Hz. This suggests that a frequency of 15 Hz may be more suiTable for preparing tooth surfaces, which might explain the less favorable results of our study for the Nd:YAG laser. Additionally, pulsed Nd:YAG lasers may treat the tooth surface more effectively than the single-pulse mode used in the present study.

Lower microleakage at the gingival margin was noted following Nd:YAG laser treatment. In the study by (23), laser beam parameters included an energy level of 50 mJ and a frequency of 15 Hz for 10 s, and a self-etch bonding agent (Clearfil SE Bond) was used. The different results of their study and ours could be attributed to the higher energy level and longer application time of the present research (100 mJ and 60 seconds, respectively). Higher energy and extended application times may cause surface degradation, negatively impacting bonding. Additionally, the use of different adhesives—total-etch in our study versus self-etch in theirs—could explain the differences. The Nd:YAG laser increases the calcium and phosphorus concentration in the dentin (23), and self-etch adhesives can chemically bond to this increased calcium, reducing microleakage. However, different results were observed due to the absence of these factors in total-etch adhesives.

According to another study, Adper Single Bond 2 formed a homogeneous hybrid layer with primary and secondary resin tags. On the other hand, when the Nd:YAG laser was used on the dentin before adhesive procedures, thinner hybrid layers and shorter resin tags were formed (24). Our study found that the microleakage was statistically comparable between Nd:YAG laser and the conventional pretreatments. But the Nd:YAG group exhibited a higher mean rank for microleakage. This finding suggests that the Nd:YAG laser may not provide the desired surface modification under the current study parameters. Differences were observed among the Er:YAG laser + Single Bond 2 and the Nd:YAG laser + Single Bond 2 and between the Er:YAG laser + Single Bond 2 and the Nd:YAG laser + Gluma at the gingival margin. The Er:YAG laser showed better results with the total-etch system than the Nd:YAG laser in the dentin, either with total-etch or universal adhesive systems. Er:YAG laser irradiation opens the dentinal tubules and creates irregular surfaces, which enhances adhesion to the dental substrate (25). In contrast, Nd:YAG radiation causes irregular melting and resolidification, which occludes some dentinal tubules. The Er:YAG laser ablates the dentin, leaving open tubules and craters, making it more effective for surface preparation (26).

The first null hypothesis was confirmed, as laser pretreatment of cavity surfaces following acid etching does not affect composite resin restorations’ microleakage. The multipurpose one-bottle universal adhesive systems represent the latest generation of adhesives and offer greater user-friendliness. These systems can be applied in different modes, providing significant advantages over previous generations by reducing the chair time and increasing flexibility in their application (27). In a study, Adper Single Bond Universal Adhesive showed more microleakage than Adper Single Bond 2, a total-etch adhesive, at the occlusal margin but less microleakage at the gingival margin (28). The pH of the universal adhesive used in their study was over 2.5, classifying it as ultra-mild. As a result, it could not sufficiently demineralize the highly mineralized enamel at the occlusal margin. In the dentin-rich gingival margin, functional monomers in the adhesive chemically bonded to the smear layer and hydroxyapatite, forming a strong bond.

In our study, the Gluma and Single Bond 2 groups had a comparable microleakage score, and the lasers did not affect Gluma adhesive efficacy. Thus, the second null hypothesis was confirmed. The 10-MDP component of universal adhesives is strong enough to chemically bond with the calcium ions remaining in hydroxyapatite (29). The pH of Gluma universal bonding is 1.6-1.8, classifying it as a moderate adhesive (30), which effectively demineralizes both enamel and dentin. Therefore, the microleakage score was comparable between the Single Bond 2 groups or those treated with Er:YAG or Nd:YAG lasers. Gluma universal adhesive seems to perform as effectively as the total-etch Single Bond 2 adhesive in Class V cavities that involve the dentin and enamel.

Numerous studies have demonstrated that microleakage is greater at the gingival margin in comparison with the occlusal margin (31,32). This disparity is likely due to the different compositions of these tissues; the dentin has a lower mineral content and a moist organic matrix, which weakens the bonding process, whereas the enamel is almost entirely mineralized (33). These findings are in the same line with the results of our study, which showed higher microleakage in the dentin than the enamel, with differences in the groups except for the Er:YAG group with Single Bond 2. This finding suggests that when the cavity margins are in the enamel, Gluma and Single Bond 2 adhesives and Er:YAG and Nd:YAG lasers can effectively prepare the cavity. The lack of difference in the Er:YAG group with Single Bond 2 likely indicates that the erbium laser effectively treats dental hard tissues when total-etch adhesive is used.

Further research is recommended to evaluate the microleakage of resin composites and adhesive systems on the tooth structure. SEM analysis should be conducted to monitor changes precisely, and different laser parameters should be tested under clinical conditions. It is also suggested that the simultaneous effects of Er:YAG and Nd:YAG lasers on universal adhesive microleakage should be investigated.

## Conclusions

The class V restorations showed higher microleakage at the gingival margin than the occlusal margin in all experimental groups except those using the Er: YAG with the Single Bond 2 adhesive. This finding suggests that the Er: YAG laser is suiTable for treating cavities in the enamel or dentin and using a total-etch adhesive. Additionally, the study indicates that the Er: YAG laser is more effective than the Nd: YAG laser at reducing the microleakage in the gingival margin.

## Figures and Tables

**Table 1:** The materials manufacture, composition, or laser parameters.

Material	Manufacturer	Composition or parameters
Nano hybrid Filtek™ Z350 XT A2 shade	3M ESPE, USA	Resin Matrix: Bis-GMA, UDMA, TEGDMA, Filler content: 78.5wt% (59.5 vol%) Silica, zirconia, aggregated zirconia/silica
Adper Single Bond 2	3M ESPE, USA	HEMA, bis-GMA, ethyl alcohol, silane-treated silica, glycerol 1,3-dimethacrylate, copolymer of acrylic and itaconic acids, diurethane dimethacrylate, water, 10% by weight of silica nanoparticles
GLUMA®Bond Universal	KULZER, Germany	4-META, MDP, Methacrylate, Acetone, Water
Nd:YAGlaser	LightWalker AT, Fotona, Ljubljana, Slovenia	Wavelength:1064nm, Power :1W, Energy:100 mj, Energy density:0.318 j/cm^2^, Pulse repetition:10HZ, Pulse duration: 60 s
Er:YAGlaser	LightWalker AT, Fotona, Ljubljana, Slovenia	Wavelength:2940nm, Power: 4W, Energy:200 mj, Energy density:0.37 j/cm^2^, Pulse repetition:20HZ, Pulse duration: 60 s

**Table 2:** Mean ± Standard Deviation of microleakage of occlusal and gingival margins among the groups.

Group	Gingival margin	Occlusal margin	P value
Single Bond 2	2.20±0.63^AB^	1.50±0.70	0.05*
Gluma Universal Bonding	2.20±0.63^AB^	1.40±0.84	0.03*
Er:YAG + Gluma Universal Bonding	1.90 ± 0.52^A^	1.33±0.51	0.03*
Er:YAG + Single Bond 2	1.80 ± 0.48^A^	1.75±0.70	0.15
Nd:YAG + Gluma Universal Bonding	2.40 ± 0.51^B^	1.75±0.74	0.01*
Nd:YAG + Single Bond 2	2.60±0.54^B^	1.80±0.44	0.01*
P value	0.004*	0.082	

Values less than 0.05 represent a significant difference between the groups according to the Wilcoxon matched-pair signed-rank. **Values less than 0.05 represent a significant difference among the groups according to the Kuskal-Wallis test. In each row, different upper-case letters represent a significant difference between the groups according to pair-wise comparison with Bonferroni correction.

## Data Availability

Not applicable.
